# Effects of complex training on physical performance in elite modern pentathletes during precompetition periods

**DOI:** 10.7717/peerj.21116

**Published:** 2026-04-20

**Authors:** Meng Liu, Zhenxiang Guo, Jun Zhou, Peng Xin, Enyi Wang, Liang Zhang, Limingfei Zhou, Yu Zhang, Bin Li

**Affiliations:** 1 School of Physical Education, University of Jinan, Jinan, China; 2 Sports Coaching College, Beijing Sport University, Beijing, China; 3 Faculty of Sport and Health Sciences, University of Jyväskylä, Jyväskylä, Central Finland Region, Finland; 4 School of Strength and Conditioning Training, Beijing Sport University, Beijing, China; 5 Qingdao Sports Training Center, Qingdao, Shandong, China; 6 Cycling and Fencing Sports Administrative Center, Under the General Administration of Sport of China, Beijing, China

**Keywords:** Complex training, Strength, Power, Agility, Modern pentathletes

## Abstract

This study compared the effects of complex training (CT) versus resistance training (RT) on agility, strength and power in elite modern pentathletes during precompetition periods. Ten male modern pentathletes from the Chinese national team participated. They first completed the RT mesocycles (eight weeks), followed by the CT mesocycles (eight weeks), with detraining microcycle (two weeks) in between for rest. Agility (three-cone test, TCT), strength (one-repetition maximum back squat, 1RM-BS; isometric mid-thigh pull peak force, IMTP), and power (counter-movement jump, CMJ; reaction strength index, RSI) performance were assessed at four time points: before RT (T0), at the end of RT (T1), before CT (T2) and at the end of CT (T3). The results revealed that the TCT improved significantly from T2 to T3 (*p* = 0.006), with a significant difference between T3 and T1 (*p* = 0.002), but no significant improvement was observed from T0 to T1 (*p* = 0.383). The 1RM-BS and IMTP improved significantly from T0 to T1 (*p* < 0.001, *p* = 0.006), from T2 to T3 (all *p* < 0.001), and T3 was significantly improved compared to T1 (all *p* < 0.001). CMJ and RSI also showed significant improvements from T0 to T1 (*p* = 0.002, *p* = 0.015), from T2 to T3 (*p* < 0.001, *p* = 0.015), and T3 was significantly better than T1 (*p* = 0.001, *p* = 0.037). These findings indicate that greater improvements in agility, strength, and power were observed during the CT mesocycle compared with the RT mesocycle in elite modern pentathletes during the precompetition period.

## Introduction

The modern pentathlon is an Olympic sport consisting of fencing, swimming, equestrian, and laser-run (cross-country running and shooting) (Le Meur et al., 2010). Agility, strength, and power are paramount for success in modern pentathlon events (Ko et al., 2021). Evidence suggests that greater agility, strength, and power capacities can improve fencing and running performance (de Vasconcelos, Cini & Lima, 2020; Pastor et al., 2022). Additionally, after the Paris 2024 Olympic Games, the equestrian riding event will be replaced by an obstacle discipline in the modern pentathlon programme, as confirmed by recent Union Internationale de Pentathlon Moderne (UIPM) and International Olympic Committee (IOC) Communications (Rappelt & Donath, 2024), further emphasizing the importance of agility, strength, and power.

The integration of resistance and plyometric training into an athlete’s training regimen is essential for enhancing maximal force and power output (MacDonald, Lamont & Garner, 2012). However, modern pentathletes need to engage in specific training sessions tailored to each event, making it challenging to incorporate regular strength training into their schedules during the precompetition period (Loureiro et al., 2022). Furthermore, both resistance and plyometric training require substantial recovery time for optimal adaptation, which is often limited during this period. As a result, sports scientists and coaches encounter difficulties in prescribing training methods that effectively induce the necessary adaptations for competitive success within the available timeframes. Complex training (CT) might offer a solution by combining resistance and plyometrics into a single session, efficiently improving both slow and fast force expression (Cormier et al., 2022). It alternates high-load resistance exercise on a set-for-set basis with an unloaded explosive exercise (Boullosa et al., 2020), and has proven particularly advantageous for female modern pentathletes to improve strength and power (Qiao et al., 2022).

CT may enhance strength and power through a combination of acute post-activation performance enhancement (PAPE) within training sessions and, more importantly, chronic neuromuscular adaptations accumulated over weeks of training. Mechanistically, PAPE is primarily attributed to increased motor unit recruitment and motor neuron excitability, as well as phosphorylation of myosin regulatory light chains, which enhances the sensitivity of the actin–myosin complex to calcium and facilitates force production at high contraction velocities (Seitz & Haff, 2016). Beyond these acute effects, the efficacy of CT can also be interpreted from a force–velocity profiling perspective. By integrating high-load resistance exercises targeting the high-force/low-velocity end of the force–velocity spectrum with plyometric or ballistic exercises emphasizing high-velocity force production, CT promotes neuromuscular adaptations across a broad range of contraction velocities. Such adaptations are particularly relevant for sports requiring rapid transitions between braking and propulsion. Moreover, CT induces stretch–shortening cycle (SSC)-specific adaptations that are critical for repeated explosive actions (Jeffreys et al., 2019). These characteristics are especially important for modern pentathlon athletes, who frequently perform SSC-dominant movements such as sprint acceleration, rapid changes of direction, and explosive take-offs during fencing, running, and the upcoming obstacle discipline. From both force–velocity and SSC perspectives, CT represents a theoretically sound and sport-specific approach for precompetition neuromuscular preparation.

Emerging evidence indicates that CT can enhance muscular strength (Jones et al., 2019), power (Pagaduan & Pojskic, 2020), and sprint capacity (Thapa et al., 2020) within relatively short and flexible training schedules. Notably, existing research in modern pentathlon has focused predominantly on female athletes (Qiao et al., 2022), whereas the effects of CT on physical performance in male elite modern pentathletes remain unclear. Given well-documented sex-related differences in strength capacity, neuromuscular characteristics, and power production, adaptations observed in female athletes may not be directly transferable to males. Moreover, considering the high demands for efficient time management during the precompetition period, it is essential to examine the effects of CT on neuromuscular performance specifically in elite male modern pentathletes.

Therefore, the purpose of this study was to evaluate the performance of elite male modern pentathletes during their precompetition preparation period after an 8-week mesocycle of CT and RT, and CT mesocycles were hypothesized to lead to greater improvements in agility, lower limb strength, and power performance than RT mesocycles.

## Methods

### Participants

An *a priori* sample size estimation was conducted using G*Power software (Heinrich-Heine-Universität, Düsseldorf, Germany). Based on a within-factor repeated-measures ANOVA design, a minimum sample size of six participants was required assuming a large effect size (*f* = 0.56) for the countermovement jump test, with an *α* error probability of 0.05 and statistical power of 0.80. The assumed effect size was informed by large within-subject effects reported in previous CT interventions in elite athletes, including studies in modern pentathlon and other power-oriented sports. Although this estimate was partly derived from prior work in female modern pentathletes, comparable effect magnitudes have been reported for strength and power outcomes following CT in trained populations. Given the applied nature of the present study and the limited availability of elite male athletes, this power analysis was intended to support study feasibility rather than to provide a definitive estimate of training effects. Ten male modern pentathletes (Mean ± SD, age: 22.60 ± 2.76 years, height: 180.56 ± 5.54 cm, weight: 74.00 ± 6.53 kg, training experience: 8.20 ± 2.20 years, BMI: 22.66 ± 0.90, body fat: 17.6 ± 1.9%, resistance training experience: 7.02 ± 1.83 years) who were preparing for the 19th Asian Games Hangzhou 2022 from the Chinese national team participated in the study. Participants had skilled resistance and plyometric training techniques. Injuries to the anterior cruciate ligament, meniscus, ankle, or any other orthopedic injury were excluded. Participant and coach informed consent was obtained before the study, and all participants and coaches signed written informed consent. The study was performed in accordance with the Declaration of Helsinki and was approved by the Ethics Committee of Sports Science Experiment and was approved by the Ethics Committee of Beijing Sport University (approval number: 2022132H).

### Experimental design and procedures

**Figure 1:**
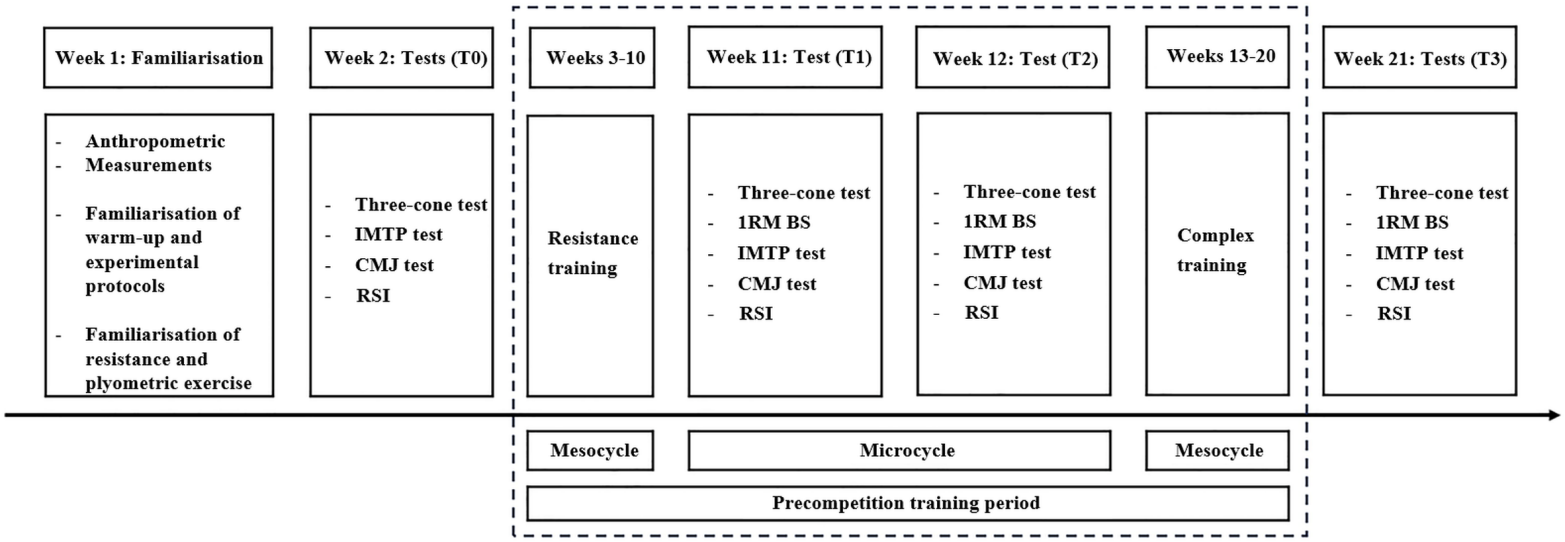
Schematic representation depicting the design and time frame of the study. 1RM-BS, one-repetition maximum back squat, IMTP, isometric mid-thigh pull, CMJ, counter-movement jump, RSI, reaction strength index.

This study adopted a longitudinal observational design with repeated within-subject measurements conducted across sequential training mesocycles during the precompetition period. Due to practical constraints associated with national team training schedules, no control group, randomization, or cross-over component was implemented (Sedgwick, 2014). The precompetition training period contained two training mesocycles and a detraining microcycle. RT mesocycles were completed first (eight weeks), followed by CT mesocycles (eight weeks). Based on linear strength training principles, each training program consisted of three phases: phase 1 aimed to improve the anatomical adaptations of muscles for one week with two sessions per week, the object of phase 2 was to enhance muscle hypertrophy for three weeks with three sessions per week, and phase 3 of four weeks with three sessions per week aimed to improve maximal strength and power. Each training session lasting 60 min. Workouts occurred on nonconsecutive days with a 48∼72 h interval between each training session. A standardized warm-up routine consisting of low-intensity running, coordination exercises, dynamic movements (lunges and skips), sprints, and dynamic stretching of the lower-limb muscles was used. After the training session, a standard cool-down including several stretching exercises (one for each muscle group involved), was performed (8–15 min). The two training mesocycles were matched for weekly training duration. Once every four weeks, the one-repetition maximum (1RM) for each movement was assessed. A two-week detraining microcycle was applied between the CT and RT training mesocycles to allow for rest for participants. Outcome measures of agility, strength, and power were assessed at time before RT (T0), the end of RT (T1), time before CT (T2), and the end of CT (T3) (Fig. 1). A 15-minute standardized warm-up was performed before each test. All tests were finished within 2 days. To avoid fatigue, participants did not perform any strenuous exercises 48 h before the test. All strength and power tests were overseen by strength and conditioning specialists certified by the National Strength and Conditioning Association (NSCA).

### Training program

The intervention consisted of a 16-week training program with RT and CT each lasting 8 weeks. The detailed training programs for RT and CT are presented in Table 1. The RT program included three weight-bearing exercises (squat, deadlift, and weight-bearing lunge), whereas the CT program paired the same resistance exercises with three plyometric exercises (squat jump, 30-cm mini-hurdle double-leg hops, and split-leg squat jump).

**Table 1:** Overview of resistance and complex training programs.

Phase	**RT (1∼8 week)**			**CT (11∼18 week)**		
	Exercises	Load (intensity × repetitions × sets)	Rest time	Exercises	Load (intensity × repetitions × sets)	Rest time
1 (1 week)	Squat	65%1RM × 15 reps × 6∼8 groups	60 s between sets	Squat + Squat Jump	(65%1RM × 15 reps + 8∼10 reps) × 3∼4 groups	30∼60 s between dynamic and plyometric exercises, and 60 s between sets
2 (3 weeks)	Deadlift	75∼80%1RM × 6 ∼12 reps × 6∼8 groups	90 s between sets	Deadlift + Mini hurdle double leg hops (30 cm)	(75 ∼80%1RM × 6∼12 reps + 6 ∼8 reps) × 3∼4 groups	30∼60 s between dynamic and plyometric exercises, and 90 s between sets
3 (4 weeks)	Weight-bearing lunge	80∼100%1RM × 1∼8 reps × 6∼8 groups	3∼4 min between sets	Weight-bearing lunge + split-leg squat jump	(80∼100%1RM × 1∼8 reps + 6∼8 reps) × 3∼4 groups	30∼60 s between dynamic and plyometric exercises, and 3∼4 min between sets

**Notes.**

1RM, 1-repetition maximum.

To ensure comparable training intensity between RT and CT, resistance loads were prescribed relative to individual strength using %1RM, and the same intensity ranges were applied across corresponding training phases in both mesocycles. Considering daily variations in training status and performance, a range of repetition numbers was allowed for each exercise (*e.g.*, 65%1RM × 15 repetitions × 6–8 sets for squat in RT), while maintaining the prescribed %1RM. To equate overall training volume/exposure between RT and CT, the number of resistance sets in CT (3–4 sets) was proportionally reduced compared with RT (6–8 sets) to compensate for the additional plyometric workload, with plyometric repetitions and recovery intervals standardized as specified in Table 1.

In addition to RT and CT, participants continued their regular sport-specific training, including technical and tactical training in fencing, swimming, and equestrian. All participants followed the same team-based sport-specific training program prescribed by the coaching staff, with identical training frequency, duration, and content throughout the intervention period. Importantly, the sport-specific training load was kept constant across the RT and CT mesocycles, and no additional individual training was allowed.

### Agility assessment

**Figure 2:**
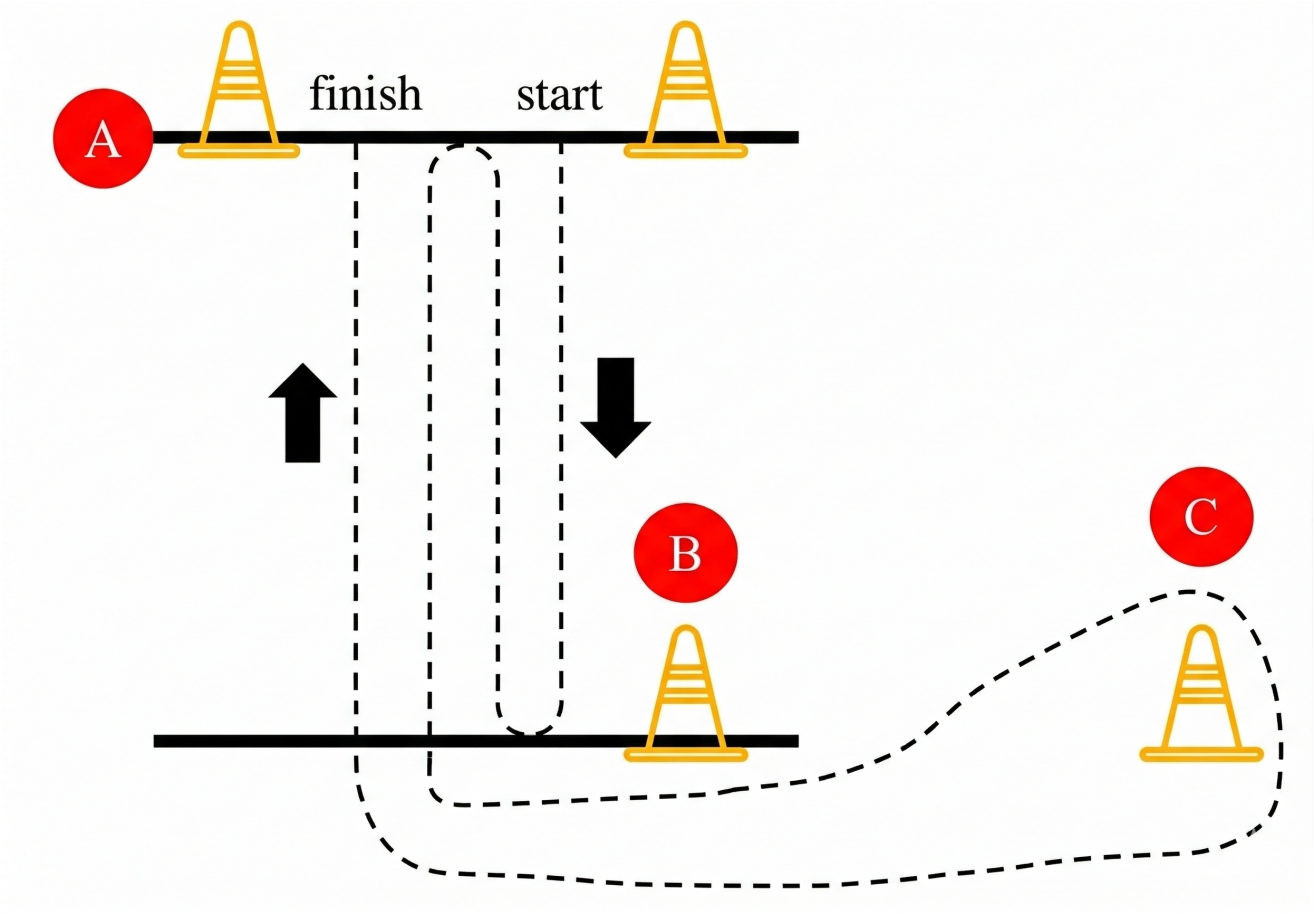
Schematic of THREE-CONE TEST.

The three-cone test was used to assess agility capacity according to the protocol previously described by Miller (2012). The three-cone test shows moderate-to-excellent test–retest reliability (ICC = 0.79, 95% CI [0.64–0.88]) (Langley & Chetlin, 2017). Participants performed two trials with maximum speed and effort, with 3 min of passive recovery between trials. Participants faced the running direction, stood on the starting line (the A-cone), and started with a standing start position. After hearing the start command “GO”, participants sprint to the B-cone, turn and sprint back to the A-cone, then turn back to the B-cone, bypassed the B-cone, and ran to the C-cone, then disregarded the C-cone and returned to the A- cone through the B-cone (Fig. 2), measurements were made using a hand-held stopwatch (S143, Seiko, Tokyo, Japan) and recorded to the nearest hundredth. The best trial, with the shortest time, was used for data analysis.

### Strength assessment

The 1RM Back Squat (1RM-BS) is a classic method of assess maximal leg strength (Miller, 2012). As a warm-up, each participant performed four sets of 10 repetitions at 20 kg, five reps at 50%, three reps at 75%, and one rep at 90% of their estimated 1RM. Following that, the load was gradually increased in steps of 5–10 kg or less until the 1RM was achieved in six attempts with a rest interval of 3 to 5 min until failure. To ensure safety, two assistants monitored each participant throughout the trial.

The Isometric Mid-thigh Pull Test (IMTP) was used to assess the isometric strength of the lower limb according to the protocol previously described by Wang et al. (2016). Each subject’s midthigh position was determined by marking the midpoint distance between the knee and hip joints before the test. Then, participants performed three dynamic warm-up sets including midthigh power cleans (one set of three repetitions) at 40%, 60%, and 80% of predetermined 1RM power clean. Following the dynamic warm-up, participants performed two isometric midthigh clean pulls at knee- and hip-joint angle. The participants were instructed to select their preferred hip and knee angle by themselves before starting the deadlift. To ensure the barbell was in contact with the mid-thigh, the height was adjusted up or down. Overhand, mixed, or hook grips were permitted. The parcitipants were instructed to pull upwards on the barbell as hard and as fast as possible and to continue their maximal effort for a duration of 6-second. To avoid precontraction, participants were instructed to relax before the command “GO!”. A rest period of 3-minute between sets was provided to ensure complete recovery. Force-time curves were recorded with a force plate (AccuPower, AMTI, Watertown, CT, USA) sampling at 1,000 Hz for each trial (Comfort et al., 2015). Participants’ peak force was defined as the highest force they achieved during the 6 s isometric test minus their body weight in Newtons. During the IMTP, participants were positioned with knee and hip angles of approximately 120–140°, consistent with commonly reported testing protocols.

### Power assessment

The height of the countermovement jump (CMJ) was used to assess lower-limb power (McLellan, Lovell & Gass, 2011). CMJ testing was performed on a force platform (Quattro Jump, Kistler, Winterthur, Switzerland) with force data sampled at 500 Hz. Participants were allowed warm-up trials and familiarization to ensure reliable performances. Participants then performed three CMJs with hands on hips, with 1 min rest between trials; countermovement depth was self-selected to maximize jump height. Take-off technique was standardized by prohibiting preliminary steps, excessive body movements, and arm swing. Flight time was determined from the force–time signal, and CMJ height was calculated from flight time using the Bosco equation (Bosco, Luhtanen & Komi, 1983). The maximum height recorded was used for analysis.

The reactive strength index (RSI) was assessed using a drop jump (DJ) to reflect the ability to rapidly transition from eccentric to concentric actions during vertical jumping (Suchomel, Sole & Stone, 2016). Participants stepped from a 45-cm box and landed on the force plate with both feet simultaneously; upon initial ground contact, they were instructed to rebound immediately into a maximal vertical jump while keeping hands on hips (Flanagan, Ebben & Jensen, 2008). Three trials were performed with 1 min rest, and the best trial was used for analysis. Ground contact time (CT) was defined as the duration from initial contact to take-off based on the vertical ground reaction force signal, and DJ height was calculated from flight time (Bosco, Luhtanen & Komi, 1983). RSI was then calculated as DJ height divided by ground contact time (Newton & Dugan, 2002). For unit consistency, jump height recorded in millimeters was converted to meters (m = mm/1,000) and contact time recorded in milliseconds was converted to seconds (s = ms/1,000), yielding RSI in m s^−^^1^:

\begin{eqnarray*}\mathrm{RSI}= \frac{\text{DJ height}}{\text{Ground contact time}} . \end{eqnarray*}

### Statistical analysis

Experimental data were processed using IBM SPSS Statistics (version 26.0, IBM Corp., Armonk, NY, USA). All data are presented as mean ± standard error (M ± SE) and 95% confidence intervals (CI). Prior to inferential analyses, the assumptions for parametric statistics were examined. Data normality was assessed using the Shapiro–Wilk test, and homogeneity of variances was evaluated using Levene’s test. All variables satisfied the assumptions for parametric analysis (*p* > 0.05). Sphericity was assessed using Mauchly’s test for all repeated-measures ANOVA models. When the assumption of sphericity was violated (*p* < 0.05), Greenhouse–Geisser corrections were applied. In the present analyses, Mauchly’s test indicated that the assumption of sphericity was met for all outcome variables; therefore, uncorrected degrees of freedom are reported. Given that the study employed a repeated-measures design, which reduces between-subject variability and increases statistical power, parametric analyses were considered appropriate despite the relatively small sample size.

A one-way repeated-measures analysis of variance (ANOVA) was conducted to examine the effect of time (T0, T1, T2, and T3) on agility (Three-cone test), strength (1RM-BS, IMTP), and power (CMJ, RSI). For variables that violated normality assumptions, the non-parametric Friedman test was applied. *Post hoc* pairwise comparisons were performed using Bonferroni adjustment to identify differences between time points, and the complete Bonferroni-adjusted *post hoc* results are provided in Table S1.

In addition to *p* values, effect sizes were reported to aid interpretation. Partial eta squared (*ηp*^2^) was calculated for repeated-measures ANOVA effects to quantify the magnitude of time effects. For within-subject pre–post changes within each training mesocycle, Cohen’s d for repeated measures was calculated using the mean difference divided by the standard deviation of the difference scores. Effect sizes were interpreted as <0.2 trivial, 0.2–0.6 small, 0.6–1.2 moderate, 1.2–2.0 large, and >2.0 very large (Hopkins et al., 2009). Statistical significance was set at *p* < 0.05 for all analyses.

## Results

**Figure 3:**
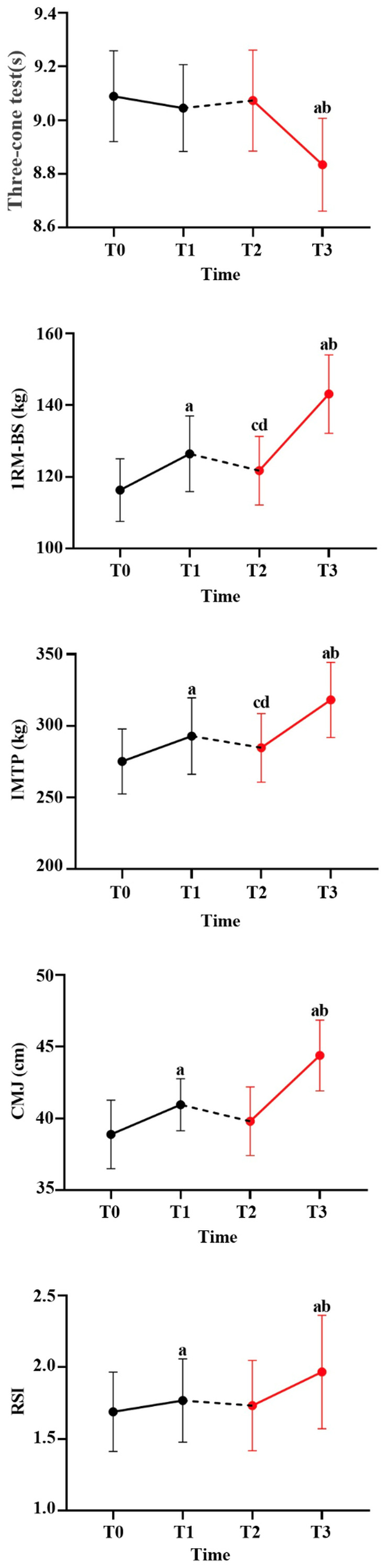
The assessment results of Three-cone test, 1RM-BS, IMTP, CMJ, and RSI after RT and CT intervention compared to all time points. (A) Significant difference from pre- to post test within the same training program (*p* < 0.05). (B) Significant difference between post test with the different training program (T3 *vs.* T1, *p* < 0.05). (C) Significant difference between T0 and T2 (*p* < 0.05). (D) Significant difference between T1 and T2 (*p* < 0.05).

Descriptive statistics (mean ± standard error) and changes in performance outcomes across the four time points (T0–T3) are presented in Table 2 and Fig. 3. All variables were normally distributed; therefore, parametric analyses were applied.

**Table 2:** The assessment results for CT and RT before and after the 8-week training mesocyles.

**Variable**	**Week 1∼8 RT mesocyle**					**Week 11∼18 CT mesocyle**				
	**T0**	**T1**	**Δ Change**	**Δ Change (%)**	***d***	**T2**	**T3**	**Δ Change**	**Δ Change (%)**	***d***
	**M**± SE (−95% CI, 95% CI)	**M**± SE (−95% CI, 95% CI)	**M**± SE (−95% CI, 95% CI)			**M**± SE (−95% CI, 95% CI)	**M**± SE (−95% CI, 95% CI)	**M**± SE (−95% CI, 95% CI)		
TCT (s)	9.09 ± 0.05 (8.97, 9.21)	9.05 ± 0.05 (8.93, 9.16)	−0.04 ± 0.21 (−0.09, 0.00)	0.44%	0.27	9.07 ± 0.06 (8.94, 9.21)	8.83 ± 0.06 (8.71, 8.96)^a^^b^	−0.24 ± 0.5 (−0.35, −0.13)	2.65%	1.32
1RM-BS (kg)	116.3 ± 2.77 (110.05, 122.56)	126.4 ± 3.33 (118.86, 133.94)^a^	10.1 ± 1.30 (7.15, 13.05)	8.68%	1.04	121.7 ± 3.03 (114.84, 133.94)^c^^d^	143.1 ± 3.46 (135.27, 150.93)^a^^b^	21.4 ± 1.70 (17.57, 25.23)	17.58%	2.08
IMTP (kg)	275.23 ± 7.18 (259, 291.47)	292.86 ± 8.43 (274.78, 311.94)^a^	17.63 ± 3.71 (9.24, 26.02)	6.41%	0.71	284.72 ± 7.56 (267.63, 301.81)^c^^d^	318.13 ± 8.28 (299.39,336.87)^a^^b^	33.41 ± 2.81 (27.04,39.78)	11.73%	1.33
CMJ (cm)	38.88 ± 0.76 (37.17, 40.59)	40.95 ± 0.57 (39.65, 42.25)^a^	2.07 ± 0.37 (1.22, 2.92)	5.32%	0.98	39.8 ± 0.76 (38.09, 41.51)	44.39 ± 0.78 (42.64, 46.16)^a^^b^	4.59 ± 0.46 (3.54, 5.64)	11.53%	1.89
RSI	1.69 ± 0.09 (1.49, 1.89)	1.77 ± 0.92 (0.66, 1.28)^a^	0.02 ± 0.15 (0.04, 0.12)	4.73%	0.28	1.73 ± 0.1 (1.51, 1.96)	1.97 ± 0.13 (1.69, 2.25)^a^^b^	0.24 ± 0.06 (0.11, 0.36)	13.87%	0.66

**Notes.**

Data are the mean ± standard error (SE) of baseline and final values and of the change on each variable in two mesocyles. Delta(Δ) change from baseline to 8 weeks, with 95% confidence interval (CI). *P* (values) for between-group comparisons between changes, adjusted for pretest values.

a Significant difference from pre- to post test within the same training program (*p* < 0.05).

b Significant difference between post test with the different training program (T3 *vs.* T1, *p* < 0.05).

c Significant difference between T0 and T2 (*p* < 0.05).

d Significant difference between T1 and T2 (*p* < 0.05).

TCT, three-cone test; 1RM-BS, one-repetition maximum back squat; IMTP, isometric mid-thigh pull; CMJ, counter-movement jump; RSI, reaction strength index.

### Agility performance

A one-way repeated-measures ANOVA revealed a significant main effect of time on agility performance assessed by the Three-cone test (*F* = 19.97, *p* < 0.001, *ηp*^2^ = 0.693). Bonferroni-adjusted *post hoc* analyses showed that Three-cone test did not change significantly during the RT mesocycle (T0 *vs.* T1, *p* = 0.383), nor between T1 and T2 (*p* = 1.000). In contrast, a significant improvement in Three-cone test was observed from T2 to T3 during the CT mesocycle (*p* = 0.006). In addition, Three-cone test at T3 was significantly lower than at T1 (*p* = 0.002), indicating a larger within-subject improvement during the CT mesocycle compared with the RT mesocycle (Table 2).

### Strength performance

For maximal strength assessed by the one-repetition maximum back squat (1RM-BS), a significant main effect of time was observed (*F* = 51.20, *p* < 0.001, *ηp*^2^ = 0.920). *Post hoc* analyses revealed significant increases in 1RM-BS from T0 to T1 during the RT mesocycle (*p* < 0.001) and from T2 to T3 during the CT mesocycle (*p* < 0.001). Furthermore, 1RM-BS at T3 was significantly greater than at T1 (*p* < 0.001), reflecting a larger within-subject gain following the CT mesocycle. A significant increase was also observed between T0 and T2 (*p* = 0.007), whereas a small but significant decrease occurred between T1 and T2 (*p* = 0.010) (Table 2).

Similarly, isometric mid-thigh pull (IMTP) peak force demonstrated a significant main effect of time (*F* = 71.42, *p* < 0.001, *ηp*^2^ = 0.892). IMTP increased significantly from T0 to T1 during the RT mesocycle (*p* = 0.006) and from T2 to T3 during the CT mesocycle (*p* < 0.001). IMTP at T3 was significantly greater than at T1 (*p* < 0.001). A significant improvement was also observed between T0 and T2 (*p* = 0.005), whereas no significant difference was found between T1 and T2 (*p* = 0.085) (Table 2).

### Power performance

For countermovement jump (CMJ) height, repeated-measures ANOVA revealed a significant main effect of time (*F* = 47.66, *p* < 0.001, *ηp*^2^ = 0.840). CMJ height increased significantly from T0 to T1 during the RT mesocycle (*p* = 0.002) and from T2 to T3 during the CT mesocycle (*p* < 0.001). CMJ at T3 was also significantly greater than at T1 (*p* = 0.001). No significant differences were observed between T0 and T2 (*p* = 0.351) or between T1 and T2 (*p* = 0.094) (Table 2).

For reaction strength index (RSI), a significant main effect of time was observed (*F* = 17.70, *p* < 0.001, *ηp*^2^ = 0.66). RSI improved significantly from T0 to T1 during the RT mesocycle (*p* = 0.015) and from T2 to T3 during the CT mesocycle (*p* = 0.015). RSI at T3 was significantly greater than at T1 (*p* = 0.037). A significant increase was also observed between T0 and T2 (*p* = 0.005), whereas no significant difference was detected between T1 and T2 (*p* = 0.085) (Table 2).

## Discussion

This study observed that (i) lower limb strength and power increased across both RT and CT mesocycles, with larger improvements occurring during the CT mesocycle, and (ii) agility performance improved significantly only during the CT mesocycle in elite male modern pentathletes during the precompetition period. These findings are consistent with previous CT research, which indicated increased limb strength (Stasinaki et al., 2015; Scott et al., 2023) power (Kyriazis et al., 2022; MacDonald et al., 2013), and agility (Cavaco et al., 2014). This suggests that CT is a promising strategy for modern pentathletes to enhance their physical performance during precompetition periods. However, these findings should be interpreted with caution because the study employed a non-randomized, fixed-order repeated-measures design (RT followed by CT), which may introduce order and time effects. In addition, repeated exposure to the performance tests could lead to learning or familiarization effects that partially confound pre–post changes

Our study utilized the THREE-CONE TEST to evaluate the agility of modern pentathletes and revealed a significant increase in the CT mesocycles compared to the RT mesocycles. The greater improvement in agility observed following the CT mesocycle may be related to neuromuscular mechanisms commonly proposed in the complex training literature. Previous studies have suggested that acute post-activation potentiation may involve increased motor neuron excitability, enhanced motor unit recruitment, and phosphorylation of myosin regulatory light chains, which collectively increase the sensitivity of the contractile apparatus to calcium (Bauer et al., 2019; Hodgson, Docherty & Robbins, 2005; Garbisu-Hualde & Santos-Concejero, 2021). However, these neuromuscular processes were not directly assessed in the present study and should therefore be interpreted as plausible, theory-driven explanations rather than confirmed physiological mechanisms underlying the observed agility adaptations.

The 1RM-BS and IMTP were used to assess lower limb muscle strength in modern pentathletes, as these indicators are crucial for the physical demands of running, swimming, and fencing in modern pentathlon (Ko et al., 2021). The results revealed that after an 8-week intervention, athletes in the CT group demonstrated a significant increase in 1RM-BS by 17.58%. A previous study also reported a similar improvement in 1RM-BS (16.88%) following an 8-week CT intervention among elite long-distance runners (Li et al., 2019). The strength gains associated with CT could be attributed to factors such as muscle morphology and neuronal adaptations (Folland & Williams, 2007). Moreover, this study observed that CT resulted in a significantly greater increase in lower limb strength compared to RT. This finding could be explained by the plyometric exercises in CT, which not only produce effects similar to resistance training on muscle hypertrophy (Grgic, Schoenfeld & Mikulic, 2021), but also provide effective neural stimulation. This stimulation may facilitate neural adaptations, such as improved motor unit recruitment patterns, firing frequency, and intermuscular coordination, as proposed in previous literature.

Muscular power performance is crucial for modern pentathletes to achieve optimal speed (Lockie et al., 2011). We utilized CMJ and RSI to assess power performance, as CMJ efficiently reflects lower limb power (Cronin & Hansen, 2005), and RSI indicates efficiency in the SSC (Jeffreys et al., 2019). Significant improvements were observed in CMJ and RSI following both CT and RT mesocycles, consistent with previous research findings (Li et al., 2019). These improvements can be attributed to the heavy weights used in both CT and RT, thereby creating favorable conditions for subsequent plyometric exercises and potential post-activation performance enhancement PAPE within training sessions (Cormier et al., 2022; Esformes & Bampouras, 2013). Additionally, our study revealed greater improvements with CT compared to RT. There is evidence to suggest that CT enhances skeletal muscle performance better than RT (Cormier et al., 2022). This advantage of CT can be attributed to its integration of high-impact plyometrics (such as squat jumps, mini hurdle double leg hops, and split-leg squat jumps), which apply maximal external force to the neuromuscular system within a short duration, leading to significant improvements in power output through enhanced leg musculotendinous stiffness. In contrast, RT involves slower isotonic muscle contractions and longer transitions between eccentric and concentric contractions (Weakley et al., 2021), which pose challenges in meeting the velocity demands of explosive exercises. Moreover, CT optimizes the force-velocity curve by combining low-load (higher-velocity) and high-load (slow-velocity) exercises to improve multiple abilities and induce a broader range of adaptive changes across the curve, which is superior to solely focusing on heavy resistance training.

A central methodological consideration of this study is the fixed-order design in which all participants completed the RT mesocycle prior to the CT mesocycle. As a result, improvements observed during the second mesocycle may partially reflect accumulated training adaptations or time-related effects rather than the isolated superiority of CT. Although a two-week detraining microcycle was implemented to reduce residual training effects, such washout periods may not fully eliminate neuromuscular adaptations accumulated over the preceding mesocycle. Therefore, the greater performance gains observed following CT should be interpreted with caution. Importantly, our analysis focused primarily on within-mesocycle pre–post changes rather than direct cross-mesocycle comparisons. While CT was associated with larger improvements in agility, strength, and power compared with RT, these findings should be viewed as indicative rather than conclusive evidence of CT superiority. It is also plausible that CT benefited from a more advanced adaptive state of the athletes following the initial RT mesocycle. Future studies employing randomized or counterbalanced designs are necessary to disentangle training-specific effects from order- or time-dependent adaptations. Accordingly, the mechanistic interpretations discussed in this study should be regarded as hypothesis-generating and grounded in existing theoretical frameworks, rather than as direct evidence of the underlying physiological mechanisms, given the absence of direct neuromuscular or biochemical measurements. But from a practical perspective, these findings provide feasible examples of how resistance and complex training can be implemented within a constrained precompetition schedule to support strength, power, and agility development in elite modern pentathletes.

Several limitations should be considered, with the most important being the non-randomized fixed-order design in which all athletes completed the RT mesocycle prior to the CT mesocycle. Consequently, performance improvements observed during the CT mesocycle may be substantially confounded by neuromuscular adaptations accumulated during the preceding RT phase, as well as by time-dependent training effects across the entire 16-week intervention period. Under this design, the greater improvements observed following CT cannot be interpreted as isolated or independent effects of complex training, but rather as adaptations occurring on top of an already enhanced neuromuscular state induced by prior RT exposure. Although a two-week detraining microcycle was implemented to attenuate residual effects, it is unlikely that such a washout period fully eliminated accumulated adaptations in elite athletes. In addition, repeated exposure to performance assessments (Three-cone test, 1RM-BS, IMTP, CMJ, and RSI) may have induced learning or test-familiarization effects, such that part of the observed improvements could reflect enhanced test execution rather than physiological adaptation alone. Standardized familiarization procedures and identical testing protocols were applied across all time points to mitigate this influence; however, learning-related effects cannot be completely ruled out. Furthermore, although all participants followed the same team-based sport-specific training program prescribed by the coaching staff and maintained consistently throughout both mesocycles, detailed quantification of sport-specific training load and session-based perceived exertion (RPE) was not systematically recorded. As a result, the relative contribution of sport-specific training, accumulated fatigue, or potentiation to the observed performance changes cannot be fully disentangled from the effects of RT or CT.

Taken together, these limitations indicate that the present findings should be interpreted as exploratory and hypothesis-generating. Future studies employing randomized or counterbalanced designs, larger samples, and systematic monitoring of sport-specific training load are required to more precisely isolate the independent effects of complex training.

## Conclusion

These findings suggest that CT may be a suitable strength-training approach for elite modern pentathletes and may provide greater improvements than RT in agility, strength, and power during the precompetition training period. The results indicate that integrating CT into precompetition training mesocycles may enhance athletic performance and could be considered a practical training strategy for coaches and practitioners.

## Supplemental Information

10.7717/peerj.21116/supp-1 Supplemental Information 1 Raw data

10.7717/peerj.21116/supp-2 Supplemental Information 2 Codebook

10.7717/peerj.21116/supp-3 Supplemental Information 3 Supplementary Table

## Additional Information and Declarations

### Data Availability

The following information was supplied regarding data availability: The raw data is available in the Supplemental File.
