# Supplementary material for: Effects of complex training on physical performance in elite modern pentathletes during precompetition periods
Source: PeerJ. 2026 Apr 20;14:e21116. doi: 10.7717/peerj.21116 (PMC13105186; doi:10.7717/peerj.21116)
Supplement: Supplemental Information 3 [file peerj-14-21116-s003.docx]

**Supplementary Table S1. Bonferroni-adjusted post hoc pairwise comparisons**

| **Outcome** | **Comparison** | **N** | **Mean Difference (I–J)** | **SE** | **95% CI** | **p (Bonferroni)** |
| --- | --- | --- | --- | --- | --- | --- |
| TCT | T0 vs T1 | 10 | 0.044 | 0.021 | -0.003, 0.091 | 0.383 |
|  | T0 vs T2 | 10 | 0.016 | 0.033 | -0.058, 0.090 | 1.000 |
|  | T0 vs T3 | 10 | 0.255 | 0.044 | 0.155, 0.355 | 0.002 |
|  | T1 vs T2 | 10 | -0.028 | 0.032 | -0.100, 0.044 | 1.000 |
|  | T1 vs T3 | 10 | 0.211 | 0.038 | 0.124, 0.298 | 0.002 |
|  | T2 vs T3 | 10 | 0.239 | 0.050 | 0.126, 0.352 | 0.006 |
| 1RM | T0 vs T1 | 10 | -10.100 | 1.303 | -13.049, -7.151 | <0.001 |
|  | T0 vs T2 | 10 | -5.400 | 1.147 | -7.995, -2.805 | 0.007 |
|  | T0 vs T3 | 10 | -26.800 | 2.065 | -31.470, -22.130 | <0.001 |
|  | T1 vs T2 | 10 | 4.700 | 1.065 | 2.291, 7.109 | 0.010 |
|  | T1 vs T3 | 10 | -16.700 | 1.868 | -20.926, -12.474 | <0.001 |
|  | T2 vs T3 | 10 | -21.400 | 1.694 | -25.233, -17.567 | <0.001 |
| IMTP | T0 vs T1 | 10 | -17.626 | 3.708 | -26.015, -9.237 | 0.006 |
|  | T0 vs T2 | 10 | -9.493 | 1.930 | -13.858, -5.128 | 0.005 |
|  | T0 vs T3 | 10 | -42.901 | 3.220 | -50.185, -35.617 | <0.001 |
|  | T1 vs T2 | 10 | 8.133 | 2.683 | 2.064, 14.202 | 0.085 |
|  | T1 vs T3 | 10 | -25.275 | 3.731 | -33.716, -16.834 | <0.001 |
|  | T2 vs T3 | 10 | -33.408 | 2.815 | -39.775, -27.041 | <0.001 |
| CMJ | T0 vs T1 | 10 | -2.070 | 0.374 | -2.916, -1.224 | 0.002 |
|  | T0 vs T2 | 10 | -0.920 | 0.425 | -1.881, 0.041 | 0.351 |
|  | T0 vs T3 | 10 | -5.510 | 0.704 | -7.102, -3.918 | <0.001 |
|  | T1 vs T2 | 10 | 1.150 | 0.387 | 0.274, 2.026 | 0.094 |
|  | T1 vs T3 | 10 | -3.440 | 0.531 | -4.642, -2.238 | <0.001 |
|  | T2 vs T3 | 10 | -4.590 | 0.464 | -5.639, -3.541 | <0.001 |
| RSI | T0 vs T1 | 10 | -0.080 | 0.019 | -0.123, -0.036 | 0.015 |
|  | T0 vs T2 | 10 | -0.043 | 0.020 | -0.089, 0.003 | 0.381 |
|  | T0 vs T3 | 10 | -0.277 | 0.054 | -0.400, -0.155 | 0.004 |
|  | T1 vs T2 | 10 | 0.036 | 0.013 | 0.007, 0.066 | 0.123 |
|  | T1 vs T3 | 10 | -0.198 | 0.056 | -0.324, -0.072 | 0.037 |
|  | T2 vs T3 | 10 | -0.234 | 0.057 | -0.363, -0.106 | 0.016 |

Pairwise comparisons between time points (T0, T1, T2, T3) for each outcome variable. Values are mean difference (I–J), standard error (SE), 95% confidence interval (CI) of the difference, and Bonferroni-adjusted p values (6 comparisons per outcome). Positive mean differences indicate higher values at the first time point.
